# Perspectives on what is needed to implement genomic medicine

**DOI:** 10.1002/mgg3.135

**Published:** 2015-03-06

**Authors:** Marc S Williams

**Affiliations:** Genomic Medicine Institute, Geisinger Health System100 N Academy Ave. Mail Stop 26-20, Danville, Pennsylvania

## Introduction

In the United States, soccer is the game of the future—and it always will be! Substitute “genomic medicine” for “soccer” and this would be an apt statement for many who criticize the amount of clinically relevant information available for the practice of medicine that has resulted from the expenditure of time and resources on the Human Genome Project and subsequent funded efforts from the National Institutes of Health (NIH) and others. The recognition of the role that NIH (particularly through the National Human Genome Research Institute [NHGRI]) had in funding research on the implementation of genomic medicine in the clinic was not explicitly stated until the release of the NHGRI's strategic plan, “Charting a course for genomic medicine from base pairs to bedside” (Green and Guyer 2011). In the subsequent years, significant progress has been made. This commentary will briefly explore the current and future state of genomic medicine emphasizing aspects that are not as frequently considered but are crucial to the successful translation of genomics into the clinic.

## Genomic Medicine: What it is and What it Isn't

In 2012, Genomic Medicine was defined by the NHGRI as, “An emerging medical discipline that involves using genomic information about an individual as part of their clinical care (e.g., for diagnostic or therapeutic decision making) and the other implications of that clinical use.” (NHGRI 2012) The tendency of some is to equate Genomic Medicine with Personalized Medicine, with the implication that care providers have been practicing “impersonalized medicine” all these years. A more useful definition of personalized medicine was put forward in the pregenome era by Pauker and Kassirer (1987), “Personalized medicine is the practice of clinical decision making such that the decisions made maximize the outcomes that the patient most cares about and minimizes those that the patient fears the most, on the basis of as much knowledge about the individual's state as is available.” This definition is particularly apt today for two reasons: It is a patient-centered definition in that it charges clinicians with understanding what the patient wants and does not want from the health care encounter and it does not give “preferred status” to any particular type of information about the patient—only insisting that we gather as much relevant information about the patient's state as needed for clinical decision making. A third concept put forward by Christensen et al. (2009) is that of Precision Medicine. The authors note that while medicine has always been personalized, as currently practiced it is intuitive meaning that care is provided for conditions that can be diagnosed only by their symptoms and only treated with therapies whose efficacy is uncertain (because evidence is based on studies of populations, not individuals) and watching for empiric response. In contrast, precision medicine is defined by the authors as the provision of care for diseases that can be precisely diagnosed, whose causes are understood, and which consequently can be treated with rules-based therapies that are predictably effective. Clearly, genomic medicine is an essential component of precision medicine and is beginning to play a role in particularly in the area of pharmacogenomics. There are numerous barriers to the full realization of genomic medicine in health care that are thoroughly discussed elsewhere (Manolio et al. 2013). A few additional issues are discussed below.

## EHRs an Essential Element for the Success of Genomic Medicine

Electronic health record systems (EHRs) are becoming ubiquitous in the hospital and clinic settings (Hsiao et al. 2011; Zhang et al. 2013). Effective use of EHRs has been shown to improve care outcomes, patient safety and care coordination with the potential for cost savings. Fully functional EHRs are capable of collecting and synthesizing data, representing knowledge around the data and returning that to the clinician in the form of point-of-care “just in time” education, and clinical decision support (CDS). This is of great importance in genomic medicine, as we know that nongenetic providers do not think they are adequately educated about genomics and the size and complexity of genomic data sets exceed human cognitive capacity. EHRs can help to overcome these barriers.

### Education

**Figure 1:**
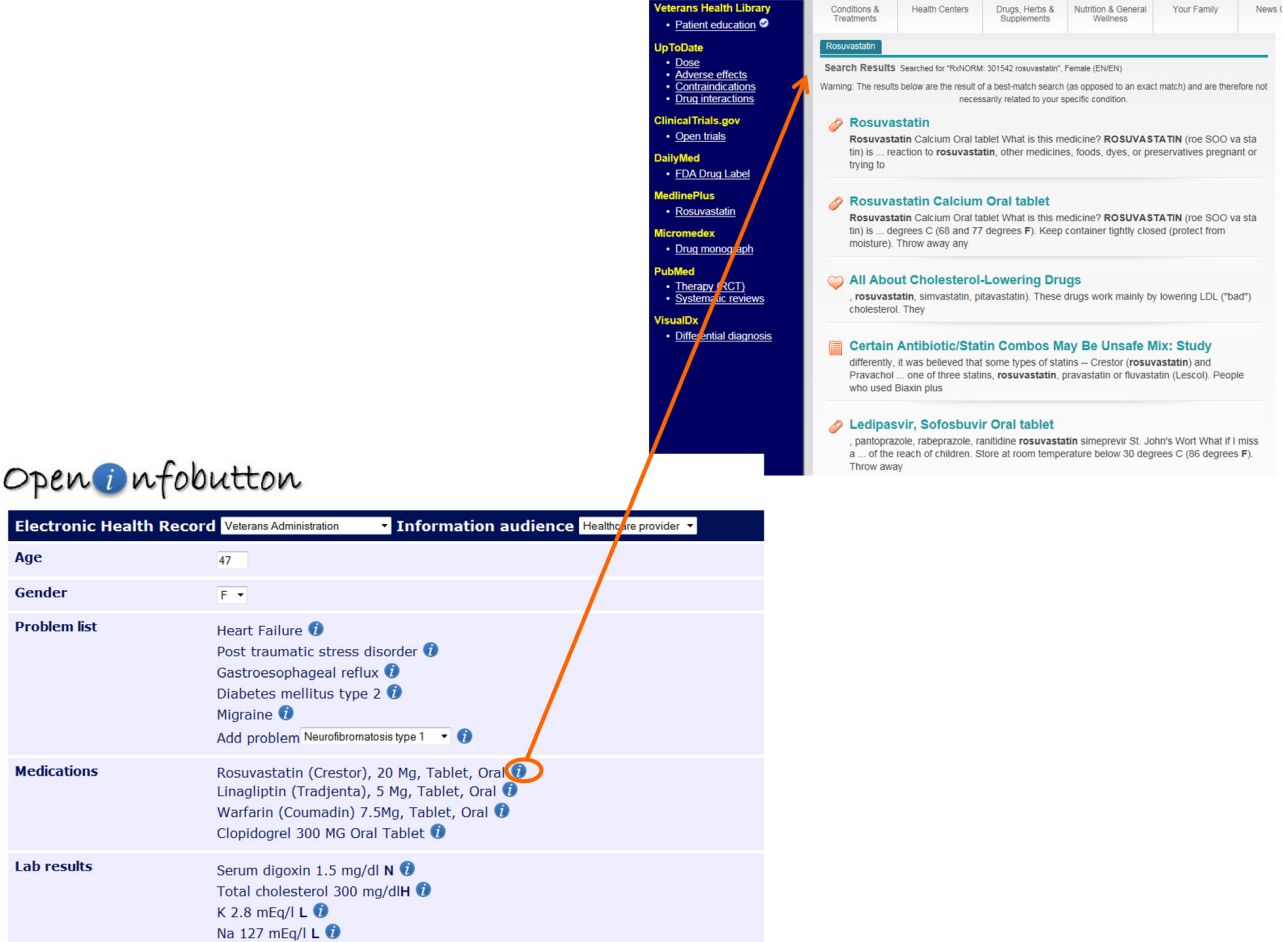
In this example infobuttons are represented as a small blue circle with an ‘i’ in the center. These can appear in many different areas of the EHR most frequently the problem list, medication list, and laboratory. Depending on the location clicking the infobutton will take the clinician to different parts of the electronic content collection. In this case, the clinician wants more information about the medication rosuvastatin, so clicks the infobutton associated with this medication. This opens a window with information about rosuvastatin obviating the need for the clinician to go to a source like Micromedex and enter rosuvastatin in the search box. This saves significant time in the clinical workflow. The infobutton uses structured information to perform these context-specific searches, in this case the RxNorm code for rosuvastatin. The navigation bar at the left allows the clinician to rapidly go to information about rosuvastatin in other content collections. Figure used with the permission of Open Infobutton and Guilherme del Fiol (Del Fiol et al. 2013; Open InfoButton Demonstration 2015).

For the most part clinicians have not been exposed to genomics in their medical training. As a result, studies have shown significant knowledge gaps in practicing physicians (Kaye and Korf 2013). EHRs can help to provide answers to genomic questions at the point of care through embedded electronic resources and infobuttons (Hoffman and Williams 2011). Electronic resources are links to content-specific knowledge repositories that are made available to clinicians in the EHR environment. Genetic resources have been linked to EHRs in a few institutions (Del Fiol et al. 2006). While the barrier to access is lowered, it still requires clinician to search the resource for information, a task that may exceed the time the clinician can spend on the task (Levy et al. 2008). Infobuttons decrease the time required to find an answer using the clinician's “location” in the EHR to provide context for the question being asked which allows the opportunity to “pre-search” content libraries. As an example if the clinician was attempting to order a medication, clicking on an infobutton would take the clinician to the resource that provided specific information about what is needed to order that specific medication as shown in Figure1. This dramatically reduces time needed to find an answer. While some genomic infobuttons have been implemented (Del Fiol et al. 2006), the emergence of infobutton standards in EHRs has led the Electronic Medical Records and Genomics (eMERGE) network and others to study the development of standards-based genomic infobuttons that could be more readily implemented across a variety of EHRs (Overby et al. 2015).

### Information for clinical decision making

The resources described in the previous section work well when the clinician recognizes an information need. However, what if the clinician does not realize there is an information need? How can information critical to making a decision be presented “just-in-time” to appropriately direct care? One solution is the use of a CDS system within EHRs. CDS “…refers broadly to providing clinicians and/or patients with clinical knowledge and patient-related information, intelligently filtered, or presented at appropriate times, to enhance patient care.” (Osheroff et al. 2007). Clinicians are familiar with one type of active CDS, alerts and reminders, in their EHRs (recognizing that familiarity breeds contempt). Too many alerts and reminders are disruptive to clinicians leading to ‘alert fatigue’ and ignoring or override which can have a negative impact on patient quality and safety. As more and more genomic information comes to bear, CDS solutions will need to go beyond alerts and reminders. As an example, the FDA black box warning states that patients with a specific genotype, HLA-B*57:01 should not receive the protease inhibitor abacavir. A CDS system today would ‘fire’ an alert for a patient with this genotype when the clinician ordered abacavir. This disrupts the physician workflow and requires them to either start over with another medication, or override the alert which could have serious consequences for the patient. An alternative would be for patients with the HLA-B*57:01 genotype abacavir would not appear on the list of medications, allowing the clinician to select a drug from the list and proceed without a stop from an alert. Only if the clinician attempted to write in abacavir would the alert fire. Most scholarship in this area is currently focused on using human factors engineering to reduce the number and increase the relevance of alerts (McCoy et al. 2014); however, research in alternatives to alerts is needed.

### The patient perspective

In the definition of personalized medicine presented above the role of the patient was emphasized. Information on a patient's preferences contextualized for specific clinical situations could be used to support shared decision making. EHRs capabilities are expanding to enhance communication between patients and providers, to accept patient generated data and comanage conditions (Newman et al. 2014). Given that germline genomic information has the potential relevance over the patient's lifetime, it is critical that the patient be engaged in the management of this information. We are studying the impact of providing genomic information to patients and families on engagement and satisfaction through the use of a genomic test report designed using input from patients (Stuckey et al. Unpublished data). Patient entered information has been used in the setting of genomic research (Do et al. 2011), but is only beginning to be piloted in the clinic.

## Implementation of Genomic Medicine

### Value is essential

The implementation of genomic medicine has been slow outside academic medical centers whose research mission often provides some incentive for innovation. Implementation will require the tools described above but will also need robust evidence regarding the value that it brings to clinical care. The concept of value is central to decision making in the health care system. Value can be thought of as a relationship between a defined set of outcomes and the costs needed to achieve those outcomes (Williams 2014). In the current environment of constrained cost, new technologies must demonstrate that they not only improve outcomes but do so at a cost that is acceptable to those responsible for paying for services. In many countries, such as the United Kingdom (UK), the assessment of value is the responsibility of a central organization such as the UK's National Institute for Health and Care Excellence. No such system exists in the United States meaning that decisions about implementing new technologies vary greatly. Institutions such as the author's must assess the value of genomic medicine from the perspective of its mission and patient population (Wade et al. 2014). There is also an emerging role for economic analysis in genomic medicine (Snyder et al. 2014) as evidenced by the NIH-funded Health Economics Common Fund Stakeholder Engagement Workshop to be held in February of 2015.

### Implementation science

Even when there is strong evidence of value, implementation of new technology in clinical care is problematic as detailed in two landmark publications by the Institute of Medicine, To Err is Human and Crossing the Quality Chasm (Kohn et al. 2000; Institute of Medicine (U.S.) 2001). Successful implementation requires not only evidence of utility and value, but an appreciation that cultural change is needed for a new intervention to be effective and sustainable. Recognition that there are common, discoverable principles associated with successful implementation led to the development of a new discipline, Implementation Science. Implementation science is defined as the scientific study of methods to promote the systematic uptake of research findings and other evidence-based practices into routine practice, and, hence, to improve the quality and effectiveness of health services (Eccles and Mittman 2006). Implementation Science has three primary aims:

1. Develop reliable strategies for improving health-related processes and outcomes and facilitate widespread adoption of these strategies
2. Produce insights and generalizable knowledge regarding implementation processes, barriers, facilitators and strategies
3. Develop, test and refine implementation theories and hypotheses including methods and measures.

The importance of the role of implementation science to genomic medicine is underscored by the explicit inclusion of implementation science methodologies for the NHGRI-funded Implementing Genomics in Practice (IGNITE) network (NHGRI 2015).

## Conclusion

While genomic medicine may have certain qualities that differ compared to current medical care, the underlying principles of personalizing care, use of electronic systems to manage increasingly large and complex sets of data, demonstration of value and implementation science are not only relevant and applicable but are essential if we are to realize the promise of genomic medicine.

## References

[b1] Christensen CM, Grossman J, Hwang J (2009). The innovator's prescription a disruptive solution for healthcare.

[b2] Del Fiol G, Williams MS, Maram N, Rocha RA, Wood GM, Mitchell JA (2006). Integrating genetic information resources with an EHR. AMIA Annu. Symp. Proc.

[b3] Del Fiol G, Curtis C, Cimino JJ, Iskander A, Kalluri AS, Jing X (2013). Disseminating context-specific access to online knowledge resources within electronic health record systems. Stud. Health Technol. Inform.

[b4] Do CB, Tung JY, Dorfman E, Kiefer AK, Drabant EM, Francke U (2011). Web-based genome-wide association study identifies two novel loci and a substantial genetic component for Parkinson's disease. PLoS Genet.

[b5] Eccles MP, Mittman BS (2006). Welcome to implementation science. Implement. Sci.

[b6] Green ED, Guyer MS (2011). National Human Genome Research Institute. Charting a course for genomic medicine from base pairs to bedside. Nature.

[b7] Hoffman MA, Williams MS (2011). Electronic medical records and personalized medicine. Hum. Genet.

[b8] Hsiao CJ, Hing E, Socey TC, Cai B (2011). Electronic health record systems and intent to apply for meaningful use incentives among office-based physician practices: United States, 2001–2011. NCHS Data Brief.

[b9] Institute of Medicine (U.S.) (2001). Crossing the quality chasm: a new health system for the 21st century.

[b10] Kaye C, Korf B (2013). Genetic literacy and competency. Pediatrics.

[b11] Kohn LT, Corrigan J, Donaldson MS (2000). To err is human: building a safer health system.

[b12] Levy HP, LoPresti L, Seibert DC (2008). Twenty questions in genetic medicine—an assessment of world wide web databases for genetic information at the point of care. Genet. Med.

[b13] Manolio TA, Chisolm RL, Ozenberger B, Roden DM, Williams MS, Wilson R (2013). Implementing genomic medicine in the clinic: the future is here. Genet. Med.

[b14] McCoy AB, Thomas EJ, Krousel-Wood M, Sittig DF (2014). Clinical decision support alert appropriateness: a review and proposal for improvement. Ochsner J.

[b15] Newman ED, Lerch V, Billet J, Berger A, Kirchner HL (2014). Improving the Quality of Care of Patients with Rheumatic Disease Using Patient-Centric Electronic Redesign Software. Arthritis Care Res. (Hoboken).

[b16] NHGRI (2012). Definition Genomic Medicine. http://www.genome.gov/pages/About/NACHGR/Sept2012AgendaDocuments/Genomic_Medicine_Definition_080112_RChisolm.pdf.

[b17] NHGRI (2015). Implementing Genomics in Practice. http://www.genome.gov/27554264.

[b18] NIH Workshop on Building a Precision Medicine Cohort. http://www.nih.gov/precisionmedicine/workshop.htm.

[b19] Open InfoButton Demonstration (2015). http://lite.bmi.utah.edu/OpenInfobuttonDemo.html.

[b20] Osheroff JA, Teich JM, Middleton BF, Steen EB, Wright A, Detmer DE (2007). A roadmap for national action on clinical decision support. J. Am. Med. Inform. Assoc.

[b21] Overby CL, Rasmussen LV, Hartzler A, Connolly JJ, Peterson JF, Hedberg RE (2015). A template for authoring and adapting genomic medicine content in the eMERGE infobutton project. JAMIA.

[b22] Pauker SG, Kassirer JP (1987). Decision Analysis. N. Engl. J. Med.

[b23] Snyder SR, Mitropoulou C, Patrinos GP, Williams MS (2014). Economic evaluation of pharmacogenomics: a value-based approach to pragmatic decision-making in the face of complexity. Public Health Genomics.

[b500] Stuckey H, Williams JL, Fan A, Rahm AK, Green J, Feldman L, Bonhag M, Zallen D, Segal MM, Williams MS Enhancing Genomic Laboratory Reports from the Patients' View: A Qualitative Analysis.

[b24] Wade JE, Ledbetter DH, Williams MS (2014). Implementation of genomic medicine in a health care delivery system: a value proposition?. Am. J. Med. Genet. C Semin. Med. Genet.

[b25] Williams MS (2014). Genomic medicine implementation: learning by example. Am. J. Med. Genet. C Semin. Med. Genet.

[b26] Zhang NJ, Seblega B, Wan T, Unruh L, Agiro A, Miao L (2013). Health information technology adoption in U.S. acute care hospitals. J. Med. Syst.

